# Efficacy of F-ACP-Containing Dental Mousse in the Remineralization of White Spot Lesions after Fixed Orthodontic Therapy: A Randomized Clinical Trial

**DOI:** 10.3390/biomedicines12061202

**Published:** 2024-05-29

**Authors:** Massimiliano Ciribè, Erika Cirillo, Martina Mammone, Giulia Vallogini, Paola Festa, Simone Piga, Gianmaria Fabrizio Ferrazzano, Angela Galeotti

**Affiliations:** 1 Dentistry Unit, Management Innovations, Diagnostics and Clinical Pathways, Bambino Gesù Children’s Hospital, IRCCS, 00165 Rome, Italy; massimiliano.ciribe@opbg.net (M.C.);; 2 Clinical Pathways and Epidemiology Unit, Medical Direction, Bambino Gesù Children’s Hospital, IRCCS, 00165 Rome, Italy; 3 UNESCO Chair in Health Education and Sustainable Development, Dentistry Section, University of Naples “Federico II”, 80138 Napoli, Italy; 4 U.N.—E.U. International Research Project on Human Health, Oral Health Section, 1200 Geneve, Switzerland

**Keywords:** white spot lesion, fixed orthodontic therapy, remineralization, caries, prevention

## Abstract

Fixed appliance (FA) therapy predisposes patients to white spot lesions (WSLs). The F-ACP complex (amorphous calcium phosphate nanoparticles enriched with carbonate and fluorine and coated with citrate) has been effective for in vitro enamel remineralization. The aim of this study was to evaluate the efficacy of the F-ACP complex in remineralizing WSLs after FA therapy. One hundred and six adolescents (aged 12–20 years) were randomized into study and control groups after FA therapy. Patients in the study group were advised to use dental mousse containing F-ACP applied within Essix retainers for six months. The presence of WSLs was recorded at baseline (T0), 3 months (T1), and 6 months (T2) according to the International Caries Detection and Assessment System (ICDAS). Visual Plaque Index (VPI) and Gingival Bleeding Index (GBI) were recorded. Among 106 study participants, 91 (52 and 39 in study and control groups, respectively) completed the study. The results showed that the ICDAS score was significantly lower (*p* < 0.001) in the study group than in the control group between T0 and T2. The application of mousse containing the F-ACP complex inside Essix retainers for six months is effective in remineralizing white spot lesions in patients after FA therapy without side effects.

## 1. Introduction

In the oral cavity, a dynamic balance exists between the hydroxyapatite minerals in the tooth structure and saliva [1]. Early carious lesions are formed when the enamel loses calcium and phosphate. These early caries are called white spot lesions (WSLs) and are non-cavitated and capable of remineralizing [2]. Fixed appliance therapy is a predisposing factor for the onset of caries and WSLs. Irregular surfaces of brackets, wires, and bands limit the natural mechanisms of salivary self-cleaning, facilitate plaque accumulation, and complicate the maintenance of good oral hygiene [3].

A rapid increase in dental plaque induces a change in microbial composition, higher levels of acidogenic bacteria such as Streptococcus mutans and Lactobacilli, and a persistently low pH for a longer period, leading to demineralization of dental enamel [4,5].

The onset of WSLs around the orthodontic attachments is one of the most common disadvantages of fixed appliance treatment. WSLs are more frequent in patients with orthodontic appliances than in those without [4] and become clinically visible approximately 4 weeks after the initiation of fixed appliance therapy in patients with poor oral hygiene [6].

Currently, there is no treatment of choice for WSLs. In recent years, several methods have been proposed to prevent the onset of WSLs, such as the professional application of topical fluoride varnish [7] and the application of mousse and varnish containing CPP-ACP (casein phosphopeptide-stabilized amorphous calcium phosphate complexes) [8,9,10] and nHAp (nano-hydroxyapatite) [11]. Moreover, microabrasion and resin infiltration have been analyzed for the non-invasive treatment of WSLs [12]. 

However, consensus on the first remineralizing therapeutic choice and the conclusions from existing systematic reviews remain controversial [13,14].

Recently, a new active substance for the remineralization of enamel was introduced: the F-ACP complex (amorphous calcium phosphate nanoparticles enriched with carbonate and fluorine and coated with citrate) contained in mousse, and the bioactive complex + HAF (hydroxyapatite partially replaced and conjugated with chitosan + hydroxyapatite partially replaced with fluoride) contained in toothpaste. 

The active substance exhibited high bioactivity and biocompatibility in in vitro studies [15,16]. Furthermore, the formulation was water-free to prevent the transformation of the amorphous phase into the inactive crystal phase. The F-ACP complex particle dissolves immediately when it comes into contact with the oral environment, in particular saliva; a release of the active ions selectively at the level of the dentin and enamel lesions follows, and a new mineral-resistant phase forms [15,16]. 

This study aimed to evaluate the efficacy of the F-ACP complex, applied inside an Essix retainer, in remineralizing white spot lesions in patients after the removal of fixed multibracket orthodontic appliances.

## 2. Materials and Methods

### 2.1. Study Design and Ethical Clearance

This was a randomized, controlled, monocenter clinical trial. The study was conducted in compliance with the latest revisions of the 1964 Helsinki Declaration, the European Commission’s 2008 Ethical Considerations for Testing of Medicinal Products on the Paediatric Population, the International Ethical Guidelines for Biomedical Research Involving Human Subjects, CIOMS-WHO (2016), and the Additional Protocol to the Oviedo Convention (2005).

### 2.2. Study Population

The study population included 106 patients aged between 12 and 20 years (51 males and 55 females; mean age ± SD = 15 ± 1.8 years) who had recently completed fixed appliance therapy not less than one month before the study timeline.

The population was randomized into two groups: study group (A) and control group (B).

#### 2.2.1. Inclusion Criteria

Patients who met the following criteria were included:

- Completion of orthodontic treatment with a fixed multibracket appliance and straight-wire technique within 1 month before the study timeline.
- Use of a removable Essix retainer for both dental arches.
- No evidence of WSLs before orthodontic therapy.
- Presence of two or more WSLs (visible with or without drying with an air/water syringe) after debonding the orthodontic appliances.
- Use of an electric toothbrush with roto-oscillating technology during the study period.
- Good general health.

#### 2.2.2. Exclusion Criteria

Those patients with the following conditions were excluded:

- Antibiotic or anti-inflammatory treatment, probiotics, or xylitol administration 1 month before enrollment in the study and during the study.
- Treatment of enamel lesions with infiltrating resins or composite materials.
- Smokers.
- Hypersensitivity to one of the components of the products.
- Presence of chronic, systemic, congenital, or degenerative diseases with limitations in manual skills to perform oral hygiene procedures independently
- Parents/patients do not agree to participate in the study.

#### 2.2.3. Screening and Randomization

A single operator screened all patients after debonding of multibrackets in fixed appliance therapy to determine their eligibility. Informed consent was obtained from the parents or legal guardians of the participants who met the inclusion criteria, and assent was obtained from the patients to be enrolled in the study.

Patients were randomized with a 1:1 allocation to the study group (A) or control group (B) according to a computer-generated randomization list, in blocks of four and layered by sex. 

During the first visit, each patient was assigned a unique randomization number associated with the following group:

- Recommendations for Group A (study group): use of toothpaste containing partially magnesium-, strontium-, carbonate-substituted hydroxyapatite conjugated with chitosan and partially fluoride-substituted hydroxyapatite (Curasept Biosmalto™ Adult Toothpaste for caries, abrasion, and erosion) for brushing teeth and impact action mousse pro containing amorphous calcium phosphate (ACP) phase functionalized with fluoride and carbonate, coated with citrate (Curasept Biosmalto™ Mousse for caries, abrasion, and erosion) inside an Essix retainer;
- Group B (control Group): Use of traditional toothpaste containing 1450 ppm of fluoride without remineralizing the active substance for brushing teeth.

### 2.3. Sample Size

The sample size for this trial was calculated based on a previous study [17] in which the remineralization of white spot lesions comparing the treated and control groups was estimated at approximately 30% on the International Caries Detection and Assessment System (ICDAS) scale (82% vs. 55% at 3 months and 78% vs. 38% at 6 months). Therefore, in the present study, we anticipated ≥30% remineralization in the study group than in the control group. The sample size was calculated for the difference in the proportion at a power of 0.9 and an α of 0.05. Therefore, the total sample size was calculated to be 70 patients (35 per group). Considering a dropout rate of 20%, 90 patients were enrolled.

### 2.4. Intervention

An intra-oral clinical examination was performed by two calibrated examiners, and the oral characteristics were recorded for all patients (Appendix A and Appendix B). The presence and location of WSLs were recorded, either on dry or wet tooth surfaces, using ICDAS (score 0–3) [2]. Furthermore, Visible Plaque Index (VPI) [18] and Gingival Bleeding Index (GBI) [19] were measured.

Photographs were obtained using a SHOFU EyeSpecial C-III Camera Device with 1:1 magnification. 

The following photographs were taken:

- a frontal intraoral photograph;
- a frontal extraoral photo;
- a photo for every tooth affected by a WSL.

For the frontal intraoral photographs, the cheeks and lips were retracted using a mouth opener to expose all 12 anterior teeth and their gums. 

Teeth affected by WSLs were photographed by positioning the camera at approximately 15° above and perpendicular to the tooth plane to minimize specular reflection and burnout. Each photograph was evaluated for quality; if it was not acceptable, the process was repeated. 

Camera settings such as exposure time, aperture, flash intensity, and e/v adjustment were determined by the investigator and maintained to take photographs of all patients at all assessment intervals.

All parameters were recorded, and photographs were taken at the beginning of the study (T0), after 3 months (T1), and after 6 months (T2). 

#### 2.4.1. Study Group (Group A)

Group A patients were instructed to use an electric toothbrush with roto-oscillating technology and a pea-sized amount of toothpaste with 1450 ppm of fluoride containing partially magnesium-, strontium-, carbonate-substituted hydroxyapatite conjugated with chitosan and partially fluoride-substituted hydroxyapatite (Curasept Biosmalto™ Adult Toothpaste for caries, abrasion, and erosion) two times a day for two minutes. The patients were then advised to wear an Essix retainer coated with impact action mousse pro containing amorphous calcium phosphate (ACP) phase functionalized with fluoride and carbonate, coated with citrate (Curasept Biosmalto™ Mousse for caries, abrasion, and erosion) after toothbrushing. The patients were instructed to apply a thickness of 3 mm of mousse inside the orthodontic retainer previously cleaned with only cold water without drying it and to remove any excess product with a finger or toothbrush to limit waste and/or ingestion of the product. The use of a hot-water rinse is contraindicated, as this may result in compromised integrity of the device. The application of the mousse inside the orthodontic retainer was recommended to extend the application time. In addition, the active substance present in the mousse was activated by cleaning the retainers. The patients used the Essix retainers on both dental arches for at least 30 min twice a day for six months.

#### 2.4.2. Control Group (Group B)

Group B patients were instructed to use an electric toothbrush with roto-oscillating technology and a pea-sized amount of traditional toothpaste with 1450 ppm of fluoride, without remineralizing the active substance, twice a day for two minutes. They were instructed to wear Essix retainers on both dental arches. 

### 2.5. Statistical Analysis

Categorical variables were summarized by absolute frequencies and percentages, and continuous variables by means and standard deviations. The Shapiro–Wilk test was used to verify the normality of the continuous data distribution. Since the treatment response items (ICDAS, VPI, and GBI) were expressed on an ordinal scale, we used Cronbach’s coefficient to evaluate the internal consistency reliability of the scale across time points [20]. At times t1, t2, and t3, the scores aggregated by group or disaggregated between groups reached reliability, assessed with a Cronbach’s alpha value of at least 0.70. To compare groups of patients, an internal consistency reliability criterion of at least 0.70 is recommended [21].

To determine the statistical differences between the groups, the chi-square or Fisher’s exact test was used for categorical variables, whereas the paired t-test or t-test for independent samples was used for continuous variables. The patients were divided into two groups according to the randomization criteria *(n* = 53 in the study group and *n* = 53 in the control group). To determine statistical differences in demographic and clinical variables between the groups at baseline, we used the chi-square test or Fisher’s exact test, and the t-test for independent samples to compare the characteristics of children with or without experimental treatment. Independent-samples t-tests were used to determine statistical differences in clinical variables between the study and control groups at three time points T0–T2, and paired t-tests were used for intra-group comparisons at three time points T0–T2. Statistical significance was set at *p* < 0.05. All statistical analyses were performed using STATA, Statistical Software: Release 13 (StataCorp LP, College Station, TX, USA).

### 2.6. Blinding and Data Management

The investigators who evaluated the data were blinded to the assignment of the participants to the study or control groups. In contrast, the operators who instructed the patients about the interventions were informed about their allocation. The data were collected anonymously from a database (Excel Version 16.85).

## 3. Results

**Figure A3:**
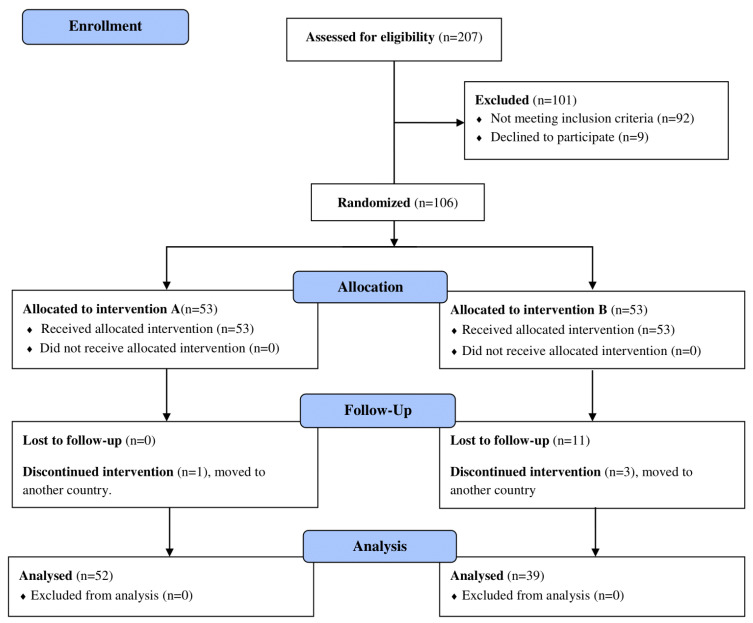
CONSORT 2010 Flow diagram.

This study initially enrolled 106 children (51 males and 55 females; age range 12–20 years; mean age ± SD = 15 ± 1.8 years). The final analysis included 91 children: 46 (50.55%) females and 45 (49.45%) males, following a 15.9% drop-out rate (15 children). A CONSORT diagram is shown in Figure A3. 

Table A1 shows the baseline demographic and clinical characteristics of the study (A) and control (B) groups at baseline (T0). The two groups were homogeneous in terms of sex and age. Table 1 shows the mean scores of the indices in the study (A) and control groups (B) at T0, T1, and T2. No statistically significant differences were observed at T0 between the groups. The ICDAS showed a statistically significant decrease (*p* < 0.05) in the study group at T1 and T2 compared with that in the control group. VPI and GBI progressively decreased at T1 and T2 in the study group; however, these data were not statistically significant.

**Figure 1:**
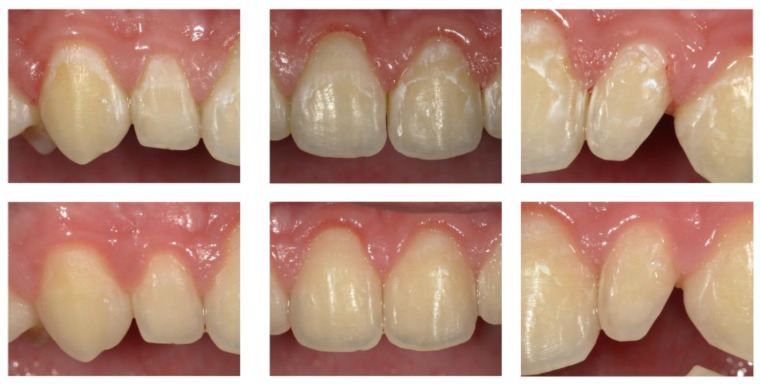
Digital intra-oral photograph obtained from a patient allocated to the study group (A) at T0 baseline and T2 after 6 months. F-ACP complex showed a remarkable decrease in lesion aspect between T0 and T2. After 6 months, in fact, no evidence of WSLs is visible after 5 s air drying.

**Figure 2:**
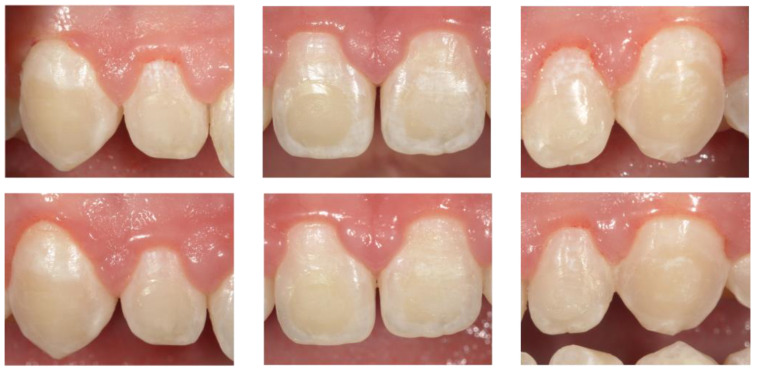
Digital intra-oral photograph obtained from a patient allocated to the control group (B) at T0 baseline, T2 after 6 months. Toothpaste with only fluoride showed little or no changes between T0 and T2. After 6 months, in fact, WSLs are still visible after 5 s air drying.

The results in Table 2 show a statistically significant reduction in ICDAS score, VPI, and GBI in comparison with the data at T0 and T2. Although all reductions were statistically significant (*p* < 0.001), a comparison of the difference scores showed that there was a larger reduction in all ICDAS indices in the study group than in the control group. Moreover, a larger reduction in lesions was clinically visible in the study group than in the control group (Figure 1 and Figure 2). 

No side effects were reported by the patients or their parents/caregivers after the application of the experimental products. 

## 4. Discussion

The present study was conducted to investigate and compare the efficacy of Curasept Biosmalto™ Adult Toothpaste for caries, abrasion, and erosion and Curasept Biosmalto™ Mousse for caries, abrasion, and erosion application on the remineralization of WSLs to that of traditional toothpaste containing fluoride only in patients after fixed appliance therapy. 

According to the American Academy of Pediatric Dentistry (AAPD), fluoride is the most effective measure for reducing dental caries prevalence in children [22]. This position is also supported by scientific literature, as evidenced by a recent meta-analysis that demonstrated that fluoride interventions are an effective measure in reducing the incidence of white spot lesions [23]. 

Currently, there is no treatment of choice for WSLs. The available treatment options can be categorized into remineralizing products and minimally invasive techniques. 

Remineralization is the first therapeutic choice for prevention and reversal of WSLs, although there is no consensus on the best remineralizing product [12]. Various enamel remineralizing protocols have been proposed in the literature, but their results remain controversial. According to published systematic reviews, the best results are obtained with a combination of two remineralizing products: fluoride toothpaste and fluoride varnish, or resin infiltration and fluoride varnish [12,13]. 

Several systematic reviews have investigated the role of casein–phosphopeptide-stabilized amorphous calcium phosphate (CPP-ACP) complexes in the prevention and remineralizing of early carious lesions [14,24,25,26]. Based on these systematic reviews, the CPP-ACP complex is effective in remineralizing WSLs; however, no statistically significant differences were observed when compared with the use of fluoride-only products or other remineralizing products. Another limitation of CPP-ACP is its potential for allergic reactions owing to the presence of casein. Therefore, individuals with allergies or intolerances to lactose and/or milk proteins may not use CPP-ACP products because they contain milk derivatives.

Our study demonstrated a statistically significant difference between the use of F-ACP and toothpaste containing only fluoride. 

Previous studies have shown the effectiveness of fluoride varnishes in reducing the WSL area and increasing its aesthetic appearance [12,27]; however, after its application, varnishes are likely to be removed easily and quickly because of the oral environment and movement of the tongue and saliva wash. In this study, we used an Essix retainer as an application tray to overcome the product dispersion limit and increase the duration of application of the remineralizing product. Furthermore, the feasibility of at-home application of F-ACP is an advantage compared to fluoride varnishes, and this approach is consequently more accessible to the patient.

Recently, nanohydroxyapatite (nHAp) has received attention because of its preventive and remineralizing properties [28]. Although the two existing systematic reviews published in 2022 [29,30] elucidated the increased remineralizing property of nano-hydroxyapatite in early caries lesions of enamel, decreased tooth hypersensitivity, and decreased surface roughness, they provided limited evidence to support its efficacy as a toothpaste component. The low number of clinical trials with relatively short follow-up periods, high risk of bias, and limited evidence does not support the efficacy of nHAp in remineralizing or preventing enamel demineralization [29,30].

In contrast, resin infiltration and microabrasion techniques were found to be the most effective treatments for the aesthetic resolution of WSLs [31] and had a significantly higher masking effect than other minimally invasive methods [32,33]. However, the surface condition, porosity, depth of the lesions, and extreme hydrophobicity of the resin reduced the efficacy of these techniques [34]. In addition, as with fluoride varnish, resin infiltration is a product intended for professional use, and the use of this technique must be the last therapeutic choice because the filling of the microporous enamel areas of WSLs would not allow any other material, such as a remineralizing product, to penetrate.

Although some RCTs on microabrasion have demonstrated the numerous clinical successes that lead to the total resolution of enamel defects [35,36], strong evidence regarding its efficacy in recent systematic reviews is lacking [31].

Our study findings showed that the high efficacy of the F-ACP complex reduces post-orthodontic WSLs more than observed with the use of fluoride toothpaste alone. Amorphous calcium phosphate plays a special role as a precursor of bioapatite (hydroxyapatite) and as a transient phase in biomineralization. The results of this study were consistent with those of previous laboratory studies that examined the remineralizing effects of F-ACP and HAF. Iafisco et al. [15] showed that F-ACP remineralizes and occludes exposed dentinal tubules after a single in vitro treatment. Ionescu et al. demonstrated through in vitro tests that F-ACP has biometric activity and that both HAF-based toothpaste and F-ACP-based mousse promoted remineralization of the tooth surface with the formation of new hydroxyapatite crystals [16].

Therefore, the results of this study highlight the importance of providing a preventive approach at the start of orthodontic treatment by first assessing the patient’s cario-receptivity, cooperation, and motivation, and suggest that the use of remineralizing products based on the F-ACP complex at the end of orthodontic treatment is beneficial for the remineralization of WSLs.

The use of the F-ACP complex may improve clinical practice as it is noninvasive but requires good adherence to therapy and home oral hygiene indications. In addition, the present study reported a reduction in oral health indices after the use of the F-ACP complex, which was often altered during and after orthodontic treatment. This evidence also highlights the importance of practicing oral hygiene instructions at home for pediatric and adolescent patients.

However, there are some study limitations: the lack of a third placebo control group and the high frequency of dropouts in the control group, which was high owing to the lack of motivation and adherence to therapy. However, the few drop-out patients in the study group suggested that the use of the F-ACP complex after orthodontic therapy was a good motivation for patients to continue post-treatment dental checkups. In addition, it should be noted that the operators who provided instructions to the study participants were not blinded to their allocation to the study or control group, which could result in bias within the study as the operators may inadvertently influence the participants’ compliance or treatment application.

## 5. Conclusions

The results of this randomized clinical trial showed that products containing the F-ACP complex were effective for the remineralization of WSLs in patients after fixed appliance therapy, and the reduction in oral health indices, such as VPI and GBI, demonstrated the importance of the patient’s motivation to practice at-home oral hygiene. Further studies are necessary to assess the long-term efficacy of the F-ACP complex in reducing WSLs over a period of six months or more and as a preventive protocol for the onset of WSLs and caries in patients planned for FA therapy. It is also recommended that the sample size be increased in order to ensure the reliability of the results. 

## Figures and Tables

**Table 1:** Mean score and standard deviation of indices in the study group (A) and in the control group (B). ICDAS (International Caries Detection and Assessment System); VPI (Visible Plaque Index); GBI (Gingival Bleeding Index); SD (standard deviation).

Parameter	Group				*p*-Value
	A (Study)		B (Control)		
	Mean	SD	Mean	SD	
ICDAS T0	1.80	0.48	1.92	0.28	0.197
ICDAS T1	1.49	0.57	1.75	0.44	0.022 *
ICDAS T2	1.00	0.56	1.53	0.65	0.001 *
VPI T0	12.05	18.36	8.06	12.44	0.250
VPI T1	10.00	14.54	12.02	28.41	0.660
VPI T2	5.25	10.68	9.03	14.28	0.146
GBI T0	9.43	17.87	8.33	14.49	0.756
GBI T1	8.05	13.71	13.19	25.30	0.202
GBI T2	3.81	8.73	7.22	14.32	0.152

* Statistically significant differences (*p* < 0.05).

**Table 2:** Mean score and standard deviation of indices in the study group (A) and in the control group (B) and comparison between times (T0 and T2). ICDAS (International Caries Detection and Assessment System); VPI (Visible Plaque Index); GBI (Gingival Bleeding Index); SD (standard deviation).

Parameter	Group A (Study)		*p*-Value	Group B (Control)		*p*-Value
	Mean	SD		Mean	SD	
ICDAS T0	1.80	0.48	<0.001 *	1.92	0.23	<0.001 *
ICDAS T2	1.00	0.56	<0.001 *	1.53	0.65	<0.001 *
difference	0.80	0.61		0.39	0.69	0.003 *
VPI T0	10.76	14.52	0.030 *	8.06	12.44	0.750
VPI T2	5.25	10.68	0.030 *	9.03	14.28	0.750
difference	5.51	15.75		−0.97	14.18	0.046 *
GBI T0	8.05	13.61	0.062	8.33	14.49	0.735
GBI T2	3.81	8.73	0.062	7.22	14.32	0.735
difference	4.23	13.70		1.11	14.50	0.293

* Statistically significant differences (*p* < 0.05).

**Figure A1:**
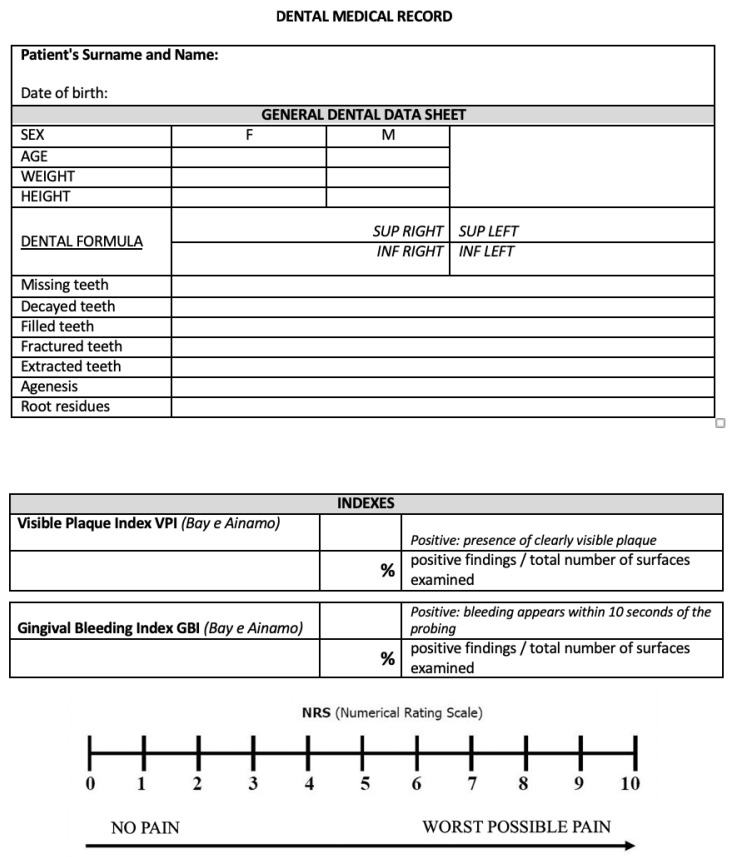
Dental medical record of oral characteristics.

**Figure A2:**
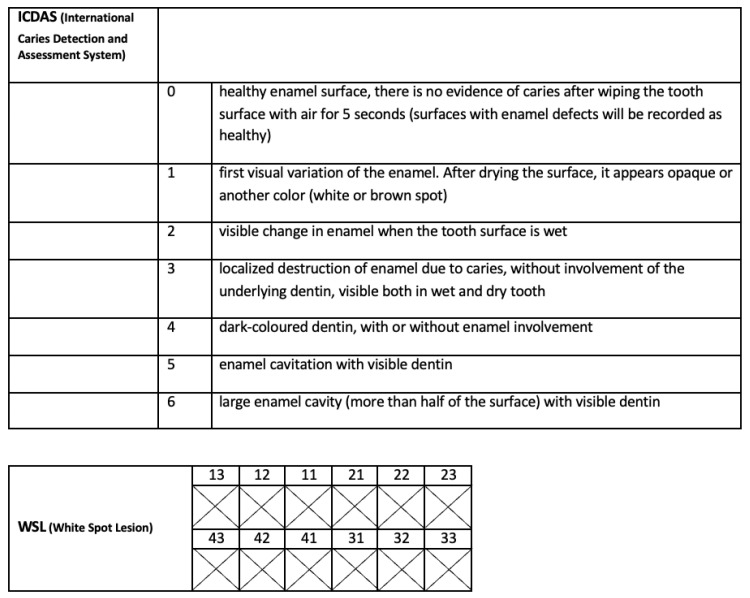
Dental medical record with ICDAS index and presence of WSLs.

## Data Availability

The protocol was registered on OpenScience Framework on 29 April 2024 (DOI: 10.17605/OSF.IO/K3WEV).

## Appendix D

**Table A1:** Demographic and clinical differences between groups at baseline, number of white spot lesions. SD (standard deviation).

	Study Group *n* = 53	Control Group *n* = 53	*p*-Value
Gender, *n* (%)			0.244
Male	26 (49.1%)	25 (47.2%)	
Female	27 (50.9%)	28 (52.8%)	
Age in years, mean (SD)	15.20 (1.85)	15.79 (1.75)	0.105
Age in years, median (range)	15 (11.25–20.22)	15 (11.72–19.68)	0.076
